# The effect of health insurance coverage on antenatal care utilizations in Ethiopia: evidence from national survey

**DOI:** 10.3389/frhs.2023.1101164

**Published:** 2023-10-06

**Authors:** Bedasa Taye Merga, Temam Beshir Raru, Alemayehu Deressa, Lemma Demissie Regassa, Mulugeta Gamachu, Belay Negash, Abdi Birhanu, Ebisa Turi, Galana Mamo Ayana

**Affiliations:** ^1^ School of Public Health, College of Health and Medical Sciences, Haramaya University, Harar, Ethiopia; ^2^ School of Medicine, College of Health and Medical Sciences, Haramaya University, Harar, Ethiopia; ^3^ Department of Public Health, Rift Valley University, Harar, Ethiopia; ^4^ Department of Public Health, Institute of Health Sciences, Wollega University, Nekemte, Ethiopia

**Keywords:** health insurance, antenatal care, prenatal care, DHS, women, Ethiopia

## Abstract

**Background:**

About three-fourths of maternal near-miss events and two-fifths of the risk of neonatal mortality can be reduced by having at least one antenatal visit. Several studies have identified potential factors related to maternal health seeking behavior. However, the association between health insurance membership and antenatal care utilization was not well investigated in Ethiopia. Therefore, this study was aimed at assessing the effect of health insurance coverage on antenatal care use in Ethiopia.

**Methods:**

The study utilized data from the 2019 Ethiopia Mini Demographic and Health Survey (EMDHS). The analysis included a weighted sample of 3,919 women who gave birth in the last five years. A logistic regression model was employed to assess the association between antenatal care use and health insurance coverage and other covariates. The results were presented as adjusted odds ratios (AOR) at a 95% confidence interval (CI). Statistical significance was declared at a *p*-value <0.05 in all analyses.

**Results:**

Antenatal care was used by 43% (95% CI: 41.46 to 44.56%) of Ethiopian women. Those with health insurance coverage had higher odds of antenatal care use than those without health insurance coverage. Women were 33% more likely to use antenatal care (ANC) if they were covered by health insurance. Age, Media access, marital status, education status, wealth index, and economic regions were also factors associated with antenatal care utilizations.

**Conclusions:**

According to our findings, less than half of Ethiopian women had four or more antenatal care visits. Health insurance membership, respondent age, media access, marital status, education status, wealth index, and economic region were factors associated with antenatal care utilization. Improving health insurance, women's economic empowerment, and education coverage are critical determinants of antenatal care utilization.

## Background

Antenatal care (ANC) can be defined as “the care provided by skilled health care professionals to pregnant women and adolescent girls in order to ensure the best health conditions for both mother and baby during pregnancy” (1). Globally, only 50% of pregnant women attended the World Health Organization (WHO) recommended minimum of four ANC visits to improve fetal and maternal health (2, 3).

Low and middle-income countries (LMIC) account for the majority (99%) of global maternal deaths. In particular, sub-Saharan Africa and Southern Asia regions bear a significant portion, contributing to over 80% of global maternal mortality (4). However, the prevalence of recommended antenatal care (ANC) utilization in these regions is low, ranging from 53.39% to 78.86% in East Africa and Southern region of Africa respectively (5).

In sub-Saharan African countries, utilizing at least one ANC visit reduces the risk of neonatal mortality by 39% (6), and a systematic review and meta-analysis found a significant effect of ANC on maternal near-miss events in Ethiopia; at least one ANC visit will avoid approximately 75% of maternal near-miss events (7).

According to a Sub-Saharan Africa study conducted from 2006 to 2018, Ethiopia had the lowest prevalence of recommended ANC utilization, at 31.88% (5). However, trends in antenatal care use (4+ ANC visits) in Ethiopia in the past 14 years showed an increment from 12% to 43%. The coverage (four or more ANC visits) has increased more than two-fold (19% to 43%) (8), since community based health insurance (CBHI) started in Ethiopia (9).

Factors affecting ANC utilizations are distance to services (10), media exposure, wealth index and availability of maternal health services (11), maternal education, women's employment, marital status, household income, cost, and pregnant women's history of obstetric complications (12, 13).

Membership to health insurance scheme also increases the odds of antenatal care utilizations (14, 15). The community-based health insurance program is widely accepted in resource limited settings as a strategy for improving healthcare utilization and health seeking behaviors and reducing out-of-pocket catastrophic payments (16) and a promising means to attain universal health coverage (17–19). In the Sustainable Development Goals (SDGs), progress on Universal Health Coverage (UHC) is tracked using two indicators: coverage of essential health services (SDG 3.8.1); and catastrophic health spending (and related indicators) (SDG 3.8.2) (20).

Universal health coverage (UHC) is a situation where every individual and all communities have unparalleled access to quality health services without suffering any form of financial hardship (21). The UHC is effective in reducing barriers to accessing essential primary care services, including antenatal care through removing financial constraints and ensuring availability, UHC enables women to seek early and regular antenatal care, leading to improved maternal and child health outcomes (10).

Several studies have identified potential factors associated with maternal health seeking behavior. However, the association between community-based health insurance coverage and antenatal care utilization was not well investigated in Ethiopia. Therefore, this study was aimed at assessing the effect of community-based health insurance coverage on antenatal care use in Ethiopia.

## Methods

### Data sources

The study utilized data from the most recent 2019 Ethiopia Mini Demographic and Health Surveys (EMDHS). The DHS is a nationwide survey collected every 5 years. The survey collects information on health and other population characteristics. The DHS employs a two-stage stratified sampling technique to collect nationally representative data from the respondents (22). At first stage sampling process begins with the selection of clusters, usually called enumeration areas (EAs). This is followed by the selection of households for the survey. The survey included a total of 8,865 participants (22). We extracted data for women who had given birth in the last five years. The analysis was conducted on a weighted sample of 3,919 women who had given birth in the last five years. Details about the DHS sampling techniques and sample size are available at http://www.dhsprogram.com/. The EMDHS research protocol complies with the National Health Research Ethics Committee and Institutional Review Board guidelines.

## Variables

### Outcome variable

The outcome variable was the number of ANC visits. The exact question in the DHS questionnaire was “how many times did you receive antenatal care during this pregnancy?” The answer was either a number or indicated they did not know. Less than four ANC visits were coded as “0” and four or more were coded as “1”. At least four ANC visits were the outcome of interest in this study.

In 2016, the WHO revised its recommendations to a minimum of eight ANC contacts, with the first contact scheduled to take place in the first trimester up to 12 weeks of gestation and subsequent contacts at 20, 26, 30, 34, 36, 38 and 40 weeks (1). However, until the end of 2021 Ethiopia hadn't implemented this new antenatal model. So, we have used the focused ANC care model which is at least 4 visits.

### Explanatory variable

The main explanatory variable was community-based health insurance coverage. The question asked was “are you covered by community-based health insurance?” Respondents answered yes or no, and since this data is binary, no further coding was needed.

### Covariates

Ten variables linked to various socio-economic and demographic indicators were added as covariates. These were economic regions (agrarians, city dwellers, and pastoralists), place of residence (urban, rural), wealth index (poorest, poorer, middle, richer, richest), family size (categorized as ≤5 members, >5 members), age of the respondent (recorded as continuous and we categorized as 15–24 years, 25–34 years, 35–49 years). Media access (no access, and has access), marital status (single, married, and widowed/separated), and education (no education, primary, and secondary and above) were covariates included in the analysis.

### Statistical analysis

Extracted data were weighted so that the sample was representative of respondents in 2019 mini EDHS. Analyses were performed using STATA version 17.0. To assess the association between antenatal care use and CBHI coverage and other covariates, a logistic regression model was employed. First, each variable was entered into a bivariable logistic regression model. Second, variables which were significant at a *p*-value of less than or equals 0.25 were fitted into a multivariable logistic regression model to identify independent factors associated with antenatal care use in Ethiopia. Statistical significance was declared at a *p*-value < 0.05 in all analyses. The results from the logistic regression analyses are presented as adjusted odds ratios (OR) with 95% confidence intervals (CIs).

## Result

### Socio-demographic characteristics of study participants

About half (50.74%) of the participants were in the age group of 25–34 years. Majority (86.6%) of the participants' household were headed by male. Nearly three-fourths of the participants were from rural settings. More than half (51.27%) of the participants had no formal education (Table 1).

**Table 1:** Socio-demographic characteristics of study participants, Ethiopia, 2019.

Variables	Frequency	Percent
Age of the respondent		
15–24	993	25.34
25–34	1,989	50.74
35–49	937	23.92
Household head sex		
Male	3,394	86.61
Female	525	13.39
Place of residency		
Urban	1,026	26.19
Rural	2,893	73.81
Educational level		
No education	2,009	51.27
Primary	1,413	36.05
Secondary and above	497	12.68
Wealth index		
Poorest	822	20.97
Poorer	820	20.92
Middle	761	19.41
Richer	704	17.98
Richest	812	20.72
Media access		
No access	2,538	64.77
Has access	1,381	35.23
Regions		
Agrarians	3,432	87.58
Pastoralists	335	8.56
City	152	3.86
Marital status		
Single	21	0.53
Married	3,677	93.83
Widowed/separated	221	5.64
Family size		
≤5 members	1,943	49.58
>5 members	1,976	50.42
CBHI membership		
Enrolled	1,186	30.26
Not enrolled	2,733	69.74

### Community-based health insurance coverage and antenatal care by socio-demographic characteristics

Only 22.1% of women with female households and 31.5% of women with male household heads were enrolled in the CBHI. Only 26.8% of women from the poorest wealth index were enrolled in the CBHI, compared to 40.3% of those from the median wealth index. About three-fifths (58.8%) of urban residents utilized ANC compared to only 37.3 of rural residents. Majority (74.4%) 0f women with secondary and higher education utilized ANC compared to only 32.4% of women with no education (Table 2).

**Table 2:** Community-based health insurance coverage and antenatal care by socio-demographic characteristics.

Variables	CBHI membership		ANC visits	
	No (%)	Yes (%)	No (%)	Yes (%)
Age of the respondent				
15–24	684 (68.9)	309 (31.1)	577 (58.1)	416 (41.9)
25–34	1,392 (70)	597 (30)	1,069 (53.8)	920 (46.2)
35–49	658 (70.2)	279 (29.8)	587 (62.6)	350 (37.4)
Household head sex				
Male	2,324 (68.5)	1,070 (31.5)	1,926 (56.7)	1,468 (43.3)
Female	409 (77.9)	116 (22.1)	307 (58.5)	218 (41.5)
Place of residency				
Urban	782 (76.2)	244 (23.8)	423 (41.2)	603 (58.8)
Rural	1,951 (67.4)	942 (32.6)	1,810 (62.6)	1,083 (37.4)
Educational level				
No education	1,401 (69.7)	608 (30.3)	1,358 (67.6)	652 (32.4)
Primary	946 (67)	467 (33)	749 (53)	664 (47)
Secondary and above	385 (77.5)	112 (22.5)	127 (25.6)	370 (74.4)
Wealth index				
Poorest	602 (73.2)	220 (26.8)	657 (79.9)	165 (20.1)
Poorer	551 (67.2)	269 (32.8)	511 (62.3)	309 (37.7)
Middle	454 (59.7)	307 (40.3)	464 (61)	297 (39)
Richer	519 (73.7)	185 (26.3)	362 (51.3)	343 (48.7)
Richest	606 (74.6)	206 (25.4)	240 (29.6)	572 (70.4)
Media access				
No access	1,728 (68.1)	810 (31.9)	1,681 (66.2)	857 (33.8)
Has access	1,005 (72.8)	376 (27.2)	553 (40)	828 (60)
Regions				
Agrarians	2,280 (66.4)	1,152 (33.6)	1,937 (56.4)	1,495 (43.6)
Pastoralists	318 (94.9)	17 (5.1)	263 (78.5)	72 (21.5)
City dwellers	135	17 (11.2)	34 (22.4)	118 (77.6)
Marital status				
Single	17 (81)	4 (19)	16 (76.2)	5 (23.8)
Married	2,562 (69.7)	1,115 (30.3)	2,067 (56.2)	1,610 (43.8)
Widowed/separated	154 (69.7)	67 (30.3)	151 (68.3)	70 (31.7)
Family size				
≤5 members	1,320 (67.9)	623 (32.1)	998 (51.4)	945 (48.6)
>5 members	1,413 (71.5)	563 (28.5)	1,235 (62.5)	741 (37.5)
CBHI membership				
Enrolled			634 (53.5)	552 (46.5)
Not enrolled			1,599 (58.5)	1,134 (41.5)

CBHI, community-based health insurance; ANC, antenatal care.

### The role of health insurance coverage on antenatal care utilizations

The antenatal care was utilized by 43% (95%CI: 41.46 to 44.56%) of Ethiopian women who had child birth in the last five years prior to the survey. Those with health insurance coverage had higher antenatal care than those without health insurance coverage (46.5% vs. 41.5%).

### Factors associated with antenatal care

In multivariable logistic regressions insurance membership, respondent age, Media access, marital status, education status, wealth index, and economic regions were factors associated with antenatal care utilizations.

The odds of antenatal care utilizations were higher among health insurance members compared to those nonmembers (AOR = 1.33, 95%CI: 1.14, 1.54). The odds of antenatal care utilizations were higher among women in the age group of 25–34 and 35–49 years compared to women aged 15–24 years (25–34 years; AOR = 1.46, 95%CI: 1.22, 1.75, 35–49 years; AOR = 1.30, 95%CI: 1.03, 1.65). Respondents who had media access had 1.36 times higher odds of antenatal care compared to those who had no media access (AOR = 1.36; 95%CI: 1.15, 1.62). The odds of antenatal care utilizations were 5.22 times higher among married women compared to the single women (AOR = 5.22; 95%: 1.72, 15.86).

The odds of antenatal care utilizations were 1.48 and 3.16 times higher among women attended primary and secondary and above education respectively compared to those who had no formal education (primary, AOR = 1.48; 95%CI: 1.26, 1.75, secondary and above AOR = 3.16; 95%CI: 2.43, 4.12). The odds of antenatal care utilizations were higher among women from higher wealth index compared to those from poorest wealth index (poorer, AOR = 2.06; 95%CI: 1.64, 2.59, middle, AOR = 1.88; 95%CI: 1.49, 2.39, richer, AOR = 2.56; 95%CI: 1.99, 3.29, richest, AOR = 4.94; 95%CI: 3.59, 6.80).

The odds of antenatal care utilizations were higher among women from higher city economic regions residence compared to those from agrarians (AOR = 1.59, 95%CI: 1.03, 2.46). The study found that respondents who were from pastoralist regions had lower odds of utilizing antenatal care compared to those from agrarians (AOR = 0.61; 95%CI: 0.45, 0.83) (Table 3).

**Table 3:** Multivariable logistic regressions of factors associated with antenatal care utilizations in Ethiopia, 2019.

ANC utilizations	COR (95% CI)	*p*-value	AOR (95% CI)	*p*-value
CBHI membership				
No	Reference			
Yes	1.23 (1.07, 1.41)	0.004	1.33 (1.14, 1.54)	0.000
Age				
15–24	Reference			
25–34	1.19 (1.02, 1.39)	0.024	1.46 (1.22, 1.75)	0.000
35–49	0.82 (0.69, 0.99)	0.044	1.30 (1.03, 1.65)	0.026
Media access				
No access	Reference			
Has access	2.93 (2.57, 3.36)	0.000	1.36 (1.15, 1.62)	0.000
Marital status				
Single	Reference			
Married	2.66 (0.95, 7.43)	0.062	5.22 (1.72, 15.86)	0.004
Widowed/separated	1.60 (0.55, 4.63)	0.388	2.69 (0.85, 8.48)	0.091
Education				
No education	Reference			
Primary	1.85 (1.60, 2.12)	0.000	1.48 (1.26, 1.75)	0.000
Secondary and above	6.09 (4.87, 7.60)	0.000	3.16 (2.43, 4.12)	0.000
Family size				
<5 members	Reference			
>5 members	0.63 (0.56, 0.72)	0.000	0.89 (0.76, 1.05)	0.172
Wealth index				
Poorest	Reference			
Poorer	2.41 (1.93, 3.01)	0.000	2.06 (1.64, 2.59)	0.000
Middle	2.55 (2.04, 3.19)	0.000	1.88 (1.49, 2.39)	0.000
Richer	3.79 (3.02,4.75)	0.000	2.56 (1.99, 3.29)	0.000
Richest	9.52 (7.58, 11.95)	0.000	4.94 (3.59, 6.80)	0.000
Economic regions				
Agrarians	Reference			
Pastoralists	0.36 (0.27, 0.47)	0.000	0.61 (0.45, 0.83)	0.002
City	4.58 (3.10, 6.76)	0.000	1.59 (1.03, 2.46)	0.037
Place of residence				
Urban	Reference			
Rural	0.42 (0.36, 0.49)	0.000	1.15 (0.93, 1.43)	0.200

COR, crude odds ratio; AOR, adjusted odds ratio; CI, confidence interval.

## Discussion

In this study, we examined the relationship between community-based health insurance coverage and ANC utilization in Ethiopia using 2019 Ethiopia mini-DHS data. One in five of women with female household heads and 31.5% of women with male household heads were enrolled in the CBHI. Only 26.8% of women from the poorest wealth index were enrolled in the CBHI, compared to 40.3% of those from the median wealth index. According to our findings, antenatal care coverage in Ethiopia is less than fifty percent. Women are 33% more likely to use ANC if they have CBHI membership. In addition to CBHI membership, the authorities should think about access to the media, education, and wealth status to improve antenatal services for Ethiopian women.

Ethiopia has been implementing CBHI since 2011 (23). The CBHI in Ethiopia targeted the rural areas and citizens mainly recruited in informal sectors such as agriculture. Its expansion in the recent time has brought a significant improvement in healthcare utilizations and financial risk protection (23).

According to EDHS 2019, 43 percent of pregnant women received prenatal care (ANC 4+) (95% CI: 41.5%–44.3%). Despite of the fact that the prevalence of ANC 4 + coverage has risen over time (12% in 2005, 19% in 2011, and 32% in 2016), more than half of pregnant women did not use it during their pregnancy. Ethiopia is ranked 35th among sub-Saharan African nations in terms of coverage of ANC, according to Ataguba JE and colleagues (24). Multi-sectorial, personal factors and community perceptions are contributing to low coverage of maternal health services in Sub-Saharan Africa. One reason for persistent low coverage of antenatal care could be related to delayed initiation of antenatal care, which hinders the completion of ANC follow-up, as the median survival time was reported was between 5 and 7 months (25, 26). Indication, community attitudes regarding cultural beliefs, the ability of decisions, and interactions with healthcare providers are potential barriers to the utilization of healthcare services during pregnancy (26–28). The uptake of antenatal care (ANC) is critical for universal reproductive coverage. ANC represents women's entrance into health services and the potential to raise safe mother and newborn targets, even though it is not expected to directly impact mortality.

The current study depicted the positive association between health insurance enrollment and ANC 4 + utilizations. Different local studies also reported a significant association (29, 30). This finding is comparable to that of previous studies conducted across Sub Saharan Africa on the association of insurance with ANC use (10, 31). The positive association might be due to insurance providing sufficient protection against catastrophic expenditure and because insurance can be linked to other socioeconomic indicators (32). Although the government provided a complete exemption for the cost of healthcare services for pregnant women, health insurance coverage is essential to eliminate out-of-pocket payments and significantly eliminate the financial barriers that often limit women's ability to attend ANC. In addition, CBHI could have other positive impacts, such as community development and local accountability of health care providers (33–37). Hence, by emphasizing the importance of CBHI and focusing on increasing enrollment, societies can remove financial barriers to healthcare access, leading to improved health-seeking behavior and better health outcomes for their populations.

Being in the highest wealth quintile increased the likelihood of ANC 4 + utilization. The finding is comparable with studies conducted in Bangladesh (38), Mozambique (39) and systematic review across SSA (40). There is strong evidence that women's economic status was a strong predictor of compliance with the nationally recommended first contact with ANC and facility delivery, with the richest women more likely to seek early health care (40–42). This positive impact of economic status could decrease the dropout rate and enable women to attend optimal ANC.

Education emerged as a significant factor in our analyses. Pregnant women with lower formal education were less likely to have ANC attendance as compared to those who had higher education level. Our study also shows that among pregnant women with formal education, those who had secondary or higher education tend to have a higher likelihood of reporting attendance at the ANC compared to their counterparts who did not have formal education. This finding is consistent as previous EDHS data report similar finding (43). Similarly, those who do not have access to the media appear to be excluded from antenatal care programs. Although health planners cannot change education levels, they should be considered in health promotion activities, using methods that are appropriate for this more susceptible demographic. In Ethiopia, health extension workers are a vital resource at the community level, and they would be used to reach women with lower levels of education. Health promotion initiatives should incorporate methods that are appropriate for this group, such as non-written tools for information, education, and communication.

## Strength and limitations of the study

The study included data from a countrywide survey, which might be viewed as strength and could improve representativeness. The limitation is that a temporal relationship between the outcome and response variables cannot be established with the cross-sectional study design.

## Conclusions

According to our findings, less than half of Ethiopian women had four and more antenatal care visits. We conclude that being covered by health insurance is associated with a higher likelihood of seeking timely ANC attendance. For this reason, we recommend that government and national health insurance authorities in Ethiopia to actively implement health insurance policies as well as roll out health educational programs that will facilitate and ensure high coverage of health insurance. Respondent age, Media access, marital status, education status, wealth index, and economic regions were also factors associated with antenatal care utilizations. Empowering the economic status of women and enhancing education coverage and media access are essential determinant to increase antenatal care utilization.

## Data Availability

The original contributions presented in the study are included in the article/Supplementary Material, further inquiries can be directed to the corresponding author.
